# Supplementary material for: Integration of parallel pathways for flight control in a hawkmoth reflects prevalence and relevance of natural visual cues
Source: eLife. 2025 Jul 17;14:RP104118. doi: 10.7554/eLife.104118 (PMC12270480; doi:10.7554/eLife.104118)
Supplement: Supplementary file 1. [file elife-104118-supp1.docx]

**Supplementary File 1**

Summary of visual stimulation conditions (labels as used in data repository <https://figshare.com/s/e680da3be83fe172a5e4>), and number of flight tracks per condition.

|  | **Condition short label** | **Condition label in  the repository** | **Condition description** | **flight track number (N)** |
| --- | --- | --- | --- | --- |
|  | N | none | no optic flow or contrast cues | 160 |
|  | G1 | right OF | right optic flow | 81 |
|  | G2 | left OF | left optic flow | 79 |
|  | G3 | lateral OF | lateral optic flow | 80 |
|  | G4 | ventral OF | ventral optic flow | 81 |
|  | G5 | dorsal OF | dorsal optic flow | 82 |
|  |  |  |  |  |
|  | S1 | ventral switch lr | ventral stripe, switch from left to right | 80 |
|  | S2 | ventral switch rl | Ventral stripe, switch from right to left | 81 |
|  | S3 | dorsal switch lr | dorsal stripe, switch from left to right | 80 |
|  | S4 | dorsal switch rl | dorsal stripe, switch from right to left | 80 |
|  | S5 | dorsal switchgrating lr | dorsal switchgrating from left to right | 174 |
|  | S6 | dorsal switchgrating lr | dorsal switchgrating from right to left | 196 |
|  |  |  |  |  |
|  | N | none2 | none2 | 156 |
|  | H1 | ventral half OF l | ventral half optic flow left | 81 |
|  | H2 | ventral half OF r | ventral half optic flow right | 82 |
|  | H3 | dorsal half long l | dorsal half parallel grating left | 87 |
|  | H6 | dorsal half long r | dorsal half parallel grating right | 82 |
|  | H7 | dorsal half OF l | dorsal half optic flow left | 81 |
|  | H4 | dorsal half OF r | dorsal half optic flow right | 88 |
|  | H8 | dorsal half OF l long r | dorsal half optic flow left parallel grating right | 203 |
|  | H5 | dorsal half long l OF r | dorsal half parallel grating left optic flow right | 126 |
|  |  |  |  |  |
|  | G6 | dorsal OF 3cm | dorsal optic flow 3cm period | 51 |
|  | G7 | dorsal OF 12cm | dorsal optic flow 12cm period | 50 |
|  | G8 | ventral OF 3cm | ventral optic flow 3cm period | 69 |
|  | G9 | ventral OF 12cm | ventral optic flow 12cm period | 52 |
|  |  |  |  |  |
|  | SG1 | dorsal switch lr right OF | dorsal stripe, switch from left to right and right optic flow | 80 |
|  | SG2 | dorsal switch rl left OF | dorsal stripe, switch right to left and left optic flow | 79 |
|  |  |  |  |  |
|  | GG1 | dorsal half OF l right OF | dorsal half optic flow left and lateral optic flow right | 126 |
|  | GG2 | dorsal half OF r left OF | dorsal half optic flow right and  lateral optic flow left | 203 |
|  |  |  |  |  |
|  | G10 | ventralOF2 | ventral optic flow 2 | 69 |
|  | G11 | dorsalOF2 | dorsal optic flow 2 | 157 |
|  | GG3 | dorsal OF ventral OF | dorsal optic flow and ventral optic flow | 227 |
|  |  |  |  |  |
|  | C1 | lateral checker | lateral random checker | 81 |
|  | SC1 | lateral checker switch lr | lateral random checker and dorsal stripe, switch from left to right | 73 |
|  | SC2 | lateral checker switch rl | lateral random checker and dorsal stripe, switch from right to left | 67 |
|  |  |  |  |  |
|  | L1 | ventral long | ventral parallel grating | 69 |
|  | L2 | dorsal long | dorsal parallel grating | 235 |
